# Biological characterization and *in vitro* fungicide screenings of a new causal agent of wheat Fusarium head blight in Tibet, China

**DOI:** 10.3389/fmicb.2022.941734

**Published:** 2022-08-05

**Authors:** Xiaoli Tang, Gongsang Yangjing, Gusang Zhuoma, Xiaofang Guo, Pengxi Cao, Benlin Yi, Wumei Wang, De Ji, Matias Pasquali, Ivan Baccelli, Quirico Migheli, Xiaoyulong Chen, Tomislav Cernava

**Affiliations:** ^1^College of Agriculture, College of Tobacco Science, Guizhou University, Guiyang, China; ^2^International Jointed Institute of Plant Microbial Ecology and Resource Management in Guizhou University, Ministry of Agriculture, China Association of Agricultural Science Societies, Guiyang, China; ^3^College of Science, Tibet University, Lhasa, China; ^4^DeFENS - Department of Food, Environmental and Nutritional Sciences, Università degli Studi di Milano, Milan, Italy; ^5^Institute for Sustainable Plant Protection, National Research Council of Italy (CNR), Sesto Fiorentino, Italy; ^6^Dipartimento Di Agraria and NRD - Nucleo di Ricerca sulla Desertificazione, Università degli Studi di Sassari, Sassari, Italy; ^7^Institute of Environmental Biotechnology, Graz University of Technology, Graz, Austria

**Keywords:** wheat Fusarium head blight, biological characterization, *Fusarium avenaceum*, Tibet, fungicide screening

## Abstract

Wheat (*Triticum aestivum* L.) is an important cereal crop, widely grown throughout the temperate zones, and also suitable for cultivation at higher elevations. Fusarium head blight (FHB) is a highly destructive disease of wheat throughout the globe. In July 2020, serious wheat FHB symptoms were observed in open fields located in Linzhi City, southeast of Tibet, China. The causal agent was identified as *Fusarium avenaceum* (Fr.) Sacc. by amplification and sequencing of the internal transcribed spacer (ITS) region, translation elongation factor 1-alpha (EF-1α) gene, and RNA polymerase II subunit (RPB-2) gene, as well as by morphological characterization. Koch’s postulates were confirmed by a pathogenicity test on healthy spikes, including re-isolation and identification. To our knowledge, this is the first report of *F. avenaceum* causing FHB on wheat in Tibet, China. Moreover, to determine pathogen characteristics that may be useful for future disease management, the utilization of different carbon and nitrogen resources, temperature, light, and ultraviolet (UV) irradiation on mycelium growth and conidia germination were studied. Soluble starch and peptone were the best carbon, and nitrogen source for the pathogen respectively. The optimal temperatures for the pathogen’s mycelium growth and conidia germination were 15–20°C, matching the average temperature during the growing season in Linzhi (Tibet). Meanwhile, alternating 8-h light and 16-h dark was shown to be conducive to mycelia growth, and complete darkness facilitated conidia germination. In addition, UV Irradiation of 48 MJ/cm^2^, approximately 100 times of the local condition, did not inhibit the germination of conidia. Furthermore, *in vitro* screening of effective fungicides was conducted. Among the seven tested pesticides, carbendazim showed the best inhibition rate, with an EC_50_ (concentration for 50% of maximal effect) value of 2.1 mg/L. Propiconazole also showed sufficient inhibitory effects against *F. avenaceum*, with an EC_50_ value of 2.6 mg/L. The study provides insights into the newly identified causal agent of wheat FHB in Tibet, China, as well as first pathogen characteristics and promising candidate substances for its management.

## Introduction

As the earliest domesticated cereal crop, *Triticum aestivum* L. was cultured by humans 10,000 years ago in the Near-Eastern Fertile Crescent. In human history, cereal crops played an important role since then by providing major nutrition sources, such as proteins, fats, minerals, vitamins, and dietary energy (Guo et al., 2020; Poole et al., 2021). Wheat is considered to have been brought to China 5000 years ago, where it became the second most important crop after rice (Wang et al., 2009; Betts et al., 2014). Nowadays, China is the largest producer and consumer of wheat in the world (CIMMYT, 2014). In Tibet, wheat is one of the main grown crops besides highland barley (*Hordeum vulgare var. coeleste* L.). The average yield of wheat in Tibet is 6–7 t/ha, well above the world average of 2.8 t/ha (Paltridge et al., 2019). Tibet is located at high altitudes where CO_2_ concentrations are relatively high; this can adversely affect the quality of wheat by restraining photosynthesis in leaves (Zhao et al., 2017). Moreover, the quality of wheat can be affected by fungal diseases as currently observed worldwide. For instance, Fusarium head blight (FHB), mainly caused by the *Fusarium graminearum* (anamorph *Gibberella zeae*) species complex (FGSC), is an economically destructive disease of wheat throughout the globe (Liang et al., 2015). Besides FGSC, *Fusarium culmorum*, and *Fusarium avenaceum* are the most common causal agents of FHB, and their geographical distribution appears to be related to environmental conditions (Wegulo et al., 2015). In the agri-food industry, FHB epidemics not only reduce grain yield and quality, but also contaminate grains with toxic secondary fungal metabolites commonly known as mycotoxins, particularly deoxynivalenol (DON) and nivalenol (NIV) (Colombo et al., 2020).

Over the last few years, the frequency of FHB epidemics has been substantially increasing worldwide. Particularly in China, long-term wheat-maize rotation, increased implementation of reduced tillage and highly sensitive wheat cultivars are the main reasons for FHB’s rapid expansion in the Yellow and Huai River Valleys (Zhu et al., 2018). Internationally, breeding wheat for resistance against FHB is a common strategy to counteract infections and disease spread (Zhu et al., 2019b). In addition, fungicides are widely screened and used to control FHB. In previous studies, triazoles (ergosterol biosynthesis inhibitors), such as prothioconazole, were shown to be effective against FHB by inhibiting the ascospore germination and mycelial growth of the *F. graminearum* (Klix et al., 2007). Some benzimidazole fungicides, like carbendazim, have been used in the control of FHB for decades (Liu et al., 2014). However, extensive and prolonged use of fungicides to control FHB has led to fungicide resistance among different *Fusarium* populations and isolates (Chen et al., 2011; Zhao et al., 2022). Except for fungicide properties, pathogen biology and environmental factors may also contribute to the development of disease, as well as fungicide resistance (Lurwanu et al., 2020; Moreno-Amores et al., 2020; Yang et al., 2021). Therefore, studying the relationship between biological characteristics of the pathogen and environmental factors is necessary. Meanwhile, screening of effective fungicides against pathogens isolated from a particular environment is needed. Tibet is known as the “roof of the world,” as well as “world’s third pole,” because it has a peculiar but also sensitive environment, which makes it a unique habitat for plants and microbes. In the past, stripe rust and powdery mildew were reported as major wheat diseases in Tibet (Li et al., 1995; Wang and Yang, 2021). However, little is known about wheat FHB occurrence and its causal agent in Tibet. Moreover, it is important to understand the biological characteristics of the pathogen as basic information of its disease epidemiology, as well as the sensitivity of local isolates to fungicides commonly used to control the disease.

In the present study, we report wheat FHB in open fields located in Linzhi city, southeast of Tibet, China. To provide the basis for future management of wheat FHB in this region, the aim of the study was to identify the causal agent, assess the responses of the pathogen to important environmental factors, and to screen effective fungicides against the pathogen *in vitro.*

## Materials and methods

### Sample collection and fungal isolation

Fusarium head blight was observed on wheat grown in open fields located in Linzhi City, Southwest of Tibet, China (29.341635°N, 94.379748°E). The attitude of the sampling site is 2913 m above the sea level. The disease incidence averaged 60–70% on a 0.7-ha field that was used for the assessment. Twelve diseased spikes with typical FHB symptoms were collected from different locations of the field. Symptomatic seeds were soaked in 4% sodium hypochlorite for 5 min, washed three times with sterilized water, placed on potato dextrose agar (PDA) medium, and incubated at 24°C. After 7 days, fungal colonies displaying morphological characteristics of *Fusarium* spp. were purified by transferring single spores. Pure cultures were obtained and selected for further identification.

### Morphological and molecular identification

A representative isolate (hereafter referred to as Charlie 779) was used for microscopic observations. Fungal conidia were observed and photographed with a Nikon Ni-E microscopic system (Nikon Inc., Melville, NY, United States). In total, 50 spores were randomly selected and measured using Nikon NIS Elements AR 4.50. For molecular identification, genomic DNA of five representative isolates was extracted from mycelia of 7-day-old cultures according to the manufacturer’s instructions (Biomiga Fungal DNA Extraction Kit; CA, United States). Then, polymerase chain reaction (PCR) amplifications of internal transcribed spacer (rDNA-ITS), elongation factor 1-alpha region (EF-1α), and RNA polymerase II subunit (RPB-2) were performed with primers ITS1 (5′-TCCGTAGGTGAACCTGCGG-3′) and ITS4 (5′-TCCTCCGCTTATTGATATGC-3′, White et al., 1990), EF1-728F (5′-CATCGAGAAGTTCGAGAAGG-3′) and TEF1L-Erev (5′-GCCATCCTTGGAGATACCAGC-3′, Carbone and Kohn, 1999), and primers RPB25F (5′-GAYGAYMGWGATCAYTTYGG-3′), and RBP2-7CR (5′-CCCATRGCTTGYTTRCCCA-3′, Liu et al., 1999), respectively. The PCR amplifications were carried out in a Bio-Rad S1000 Thermal Cycler in a 25 μl reaction mixture containing 2 × Taq PCR MasterMix (Sangon, Inc.), 1 μl DNA template and 1 μl of each primer. The PCR conditions were as follows: initial denaturation at 94^°^C for 3 min, followed by 35 cycles of denaturation at 95^°^C for 30 s, annealing for 30 s at the corresponding temperatures (53^°^C for ITS, 60^°^C for EF-1α gene, and 55^°^C for RPB2), extension at 72^°^C for 45 s, then a final extension for 10 min.

The obtained DNA sequences in this study were queried against the National Center Biotechnology Information (NCBI) database with other DNA sequences in the GenBank database. Representative sequences of the sequenced DNA regions were deposited in GenBank. In addition, a phylogenetic tree was constructed with MEGA 7 based on ITS region, EF1-α gene and RPB2 gene sequences.

### Pathogenicity test

Koch’s postulates were met to confirm the pathogenicity of *F. avenaceum* Charlie 779 on wheat cv. Zangdong 16 at the flowering stage in a glasshouse assay. The spikes of 20 wheat plants were each sprayed with 500 μL conidia suspension (1 × 10^3^ spores/ml). Twenty plants that were mock-inoculated with sterilized water served as the controls. All plants were incubated at 26 ± 2°C under a 16 h/8 h photoperiod and 70–75% relative humidity (RH) after inoculation.

### Utilization of different carbon and nitrogen sources

The mycelium growth and conidia germination rate of *F. avenaceum* Charlie 779 on different carbon sources and nitrogen sources were determined in Czapek’s medium by implementing methods that were adapted from previous studies (Song et al., 2020; Li et al., 2021). In total, five carbon sources were tested, soluble starch, D-mannose, glucose, maltose, and D-Levulose, with no carbon source as control. In complementary assessments, five nitrogen sources were tested, including peptone, sodium nitrate, L-arginine base, beef extract, and ammonium sulfate, with no nitrogen source as control. For each treatment, three replications were conducted. To study the mycelial growth on the different carbon and nitrogen sources, a seven-day-old colony was placed in the center of a Petri dish containing the respective medium and then incubated at 28°C for 5 d under dark conditions. For the germination rate analysis, conidia from 10-day-old colonies were washed with distilled water and their concentrations were adjusted to 1.0 × 10^5^ conidia mL^–1^ using a hemocytometer. Then, 0.2 mL conidia suspension was added to 0.2 mL Czapek broth complemented with different carbon and nitrogen sources. After 12 h, the germination rate was evaluated by microscopy at 100 times magnification.

### The influence of temperature on mycelium growth and conidia germination rate

The influence of temperature on mycelium growth and conidia germination rate was studied on PDA and Potato Dextrose Broth (PDB) medium, respectively, incubated at temperatures ranging from 15 to 35°C at 5°C intervals. A seven-day-old colony was placed in the center of a Petri dish with PDA medium and then incubated at different temperatures. The mycelial growth was recorded at 5 days post inoculation. The conidia germination rate in PDB medium under different temperatures was calculated.

### The influence of photoperiod on mycelium growth and conidia germination rate

To study the effect of the photoperiod, inoculated growth medium (PDA for mycelium growth, and PDB for conidia germination rate) was placed in incubators at 28°C with following settings: (i) 24 h light; (ii) 24 h dark; (iii) 8 h light and 16 h dark; (IV) 12 h light and 12 h dark. Each treatment was conducted with three replicates.

### The influence of ultraviolet irradiation on mycelium growth and conidia germination rate

After growth on PDA at 28°C for 10 days, conidia suspensions were prepared. The cell density of the suspensions was adjusted to 1.0 × 10^4^ colony-forming units (CFU)/mL, and 50 μL were pipetted per plate. Open plates were irradiated by using a UV lamp that was mounted 40 cm from the plates under sterile conditions. The irradiation was performed using UVC (Haier, China) at a wavelength of 254 nm for five different durations, including 0 (control group), 1, 5, 10 and 20 min. The plates were then incubated at 28°C for 4–5 days and subjected to regular examination to record the number of fungal colonies.

### *In vitro* antifungal activity of fungicides on mycelial growth

The mycelial growth rate method was used to evaluate antifungal activity of fungicides (Xin et al., 2020). Different fungicides were dissolved in organic solvents or water (propiconazole and pyrimethanil were dissolved in ethanol and myclobutanil was dissolved in acetone, all other fungicides were dissolved in water), and then mixed with the PDA medium at different concentrations (Table 1). For these fungicides dissolved in either ethanol or acetone, a comparable final concentration of the indicated solvents was added into PDA medium, which served as control plates (CK). Afterward, a *F. avenaceum* colony (with a diameter of 6 mm) was placed in the center of the PDA medium-containing plates and cultured at 28°C and 75% RH for 7 day under dark conditions. Then the colony diameter (mm) was measured using a ruler. The EC_50_ (concentration for 50% of maximal effect) values of different fungicides were calculated using DPS v2.0 software.

**TABLE 1:** Concentrations of substances used for fungicide sensitivity assays and their China pesticide registration numbers (http://www.chinapesticide.org.cn).

Fungicide name	Registration number	Active ingredient	Substance concentration (μg/mL)
40% propiconazole EC	PD20093418	propiconazole	100, 50, 10, 5, 1
12.5% myclobutanil EC	PD20086370	myclobutanil	100, 50, 10, 5, 1
40% pyrimethanil SC	PD20060014	pyrimethanil	1000, 500, 250, 125, 62.5
50% thiram WP	PD20093058	thiram	500, 300, 150, 30, 15
80% mancozeb WP	PD20082030	mancozeb	300, 100, 50, 25, 12.5
50% carbendazim WP	PD85150-35	carbendazim	6, 3, 1.5, 0.75, 0.38
70% hymexazol WP	PD20100877	hymexazol	200, 100, 50, 25, 12.5

The employed fungicides were formulations of propiconazole (40% Emulsifiable Concentrates) (EC), myclobutanil (12.5% EC), pyrimethanil (40% Suspension Concentrate) (SC), Thiram (50% Wettable Powder) (WP), mancozeb (80% WP), carbendazim (50% WP), and hymexazol (70% WP) and were purchased from local distributors.


$$\text{I}=\frac{\text{C}-\text{T}}{\text{C}}\times 100$$

Equation used to determine inhibition efficiency of the fungicides. I: percent inhibition, C: radial growth of the fungus in the control, T: radial growth of the fungus in the treatment.

## Results

### Morphological characterization

**FIGURE 1:**
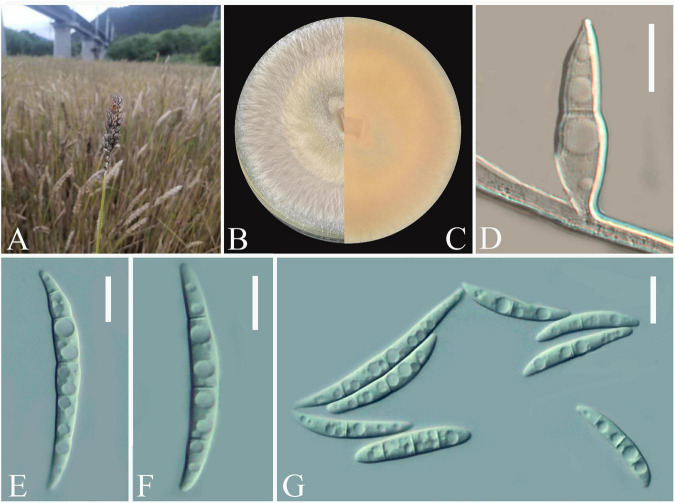
Open field symptoms of wheat Fusarium head blight **(A)**, colony of *Fusarium* sp. Charlie 779 on potato dextrose agar (PDA) medium **(B,C)**, macroconidia of *Fusarium* sp. Charlie 779 on septate mycelium **(D)**, macroconidia of *Fusarium* sp. Charlie 779 **(E–G)**. Scale bars: D–G = 10 μm.

Twenty-one *Fusarium-*like isolates were obtained from diseased wheat spikes (Figure 1A). The colonies were white with a regular circular shape at the early stage and gradually became fluffy after 7 days (Figure 1B). Pure cultures of all isolates were stored in the fungal pathogen inventory of Guizhou Provincial key Laboratory for Agricultural Pest Management of the Mountainous Region (accession no. GZUPP:1450). A strong pink pigmentation was observed on the reverse side of the PDA plates. For microscopic observations, one typical strain *Fusarium* sp. Charlie 779 was selected from all obtained isolates. Conidia were solitary, macrospores slender, straight to slightly falcate with 3 to 5 septa, and ranged from 30.2–60.8 × 3.9–5.4 μm (*n* = 50) (Figures 1D–G). Based on these morphological characteristics, the isolates matched the description of the genus *Fusarium* (Nelson et al., 1983).

### Molecular characterizations

**FIGURE 2:**
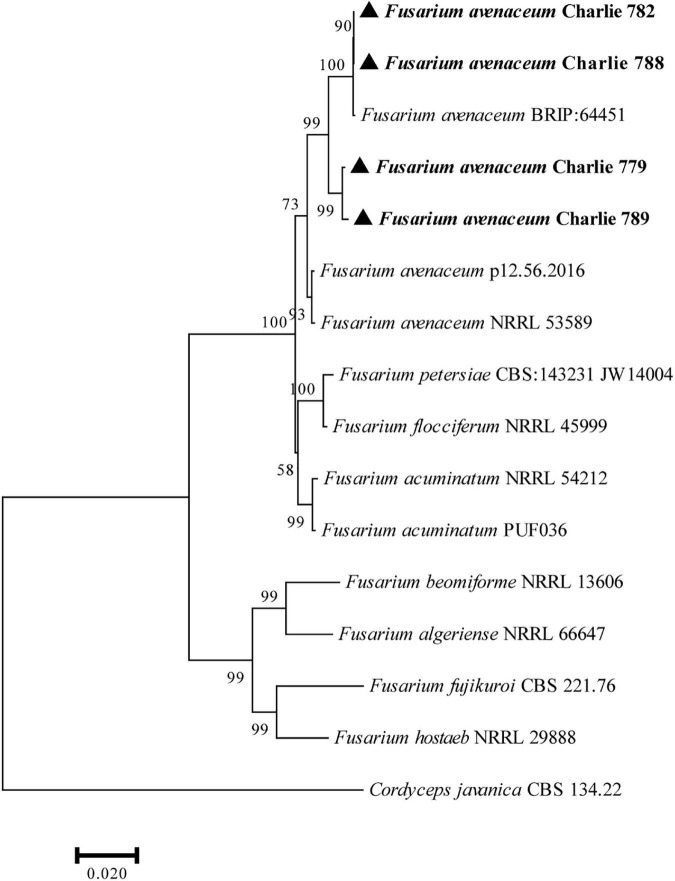
Phylogenetic analysis of concatenated sequences of the ITS region, EF-1α and RPB-2 obtained from *F. avenaceum* Charlie 779, *F. avenaceum* Charlie 782, *F. avenaceum* Charlie 788, and *F. avenaceum* Charlie 789 from this study and reference sequences of *Fusarium* spp. specimens using the maximum likelihood method (1000 bootstrap iterations). *Cordyceps javanica* CBS 134.22 was used as the outgroup. Bootstrap values are provided next to the respective branches.

The obtained DNA sequences were compared with other DNA sequences following alignment with the GenBank. BLAST analysis of the sequenced fragments resulted in the best match to *F. avenaceum* sequences (ITS, 99.36% identity to MF687287.1; EF-1α, 99.60% identity to MK836081.1; RPB2, 98.79% identity to JX171663.1). Representative sequences of the sequenced DNA regions were deposited in GenBank (Charlie 779, accession numbers: ITS, MZ049674; RPB-2, MZ053464; EF-1α, MZ053465; Charlie 782, accession numbers: ITS, ON805846; RPB-2, ON833471; EF-1α, ON833468; Charlie788, accession numbers: ITS, ON805862; RPB-2, ON833472; EF-1α, ON833469; Charlie 789, accession numbers: ITS, ON819345; RPB-2, ON833473; EF-1α, ON833470; Charlie 790, accession numbers: ITS, ON847358; RPB-2, ON868917; EF-1α, ON868916) and are included in the Supplementary Material. Furthermore, a phylogenetic tree was constructed with MEGA 7 based on combined sequences of the ITS region together with EF-1α and RPB-2 genes by using the maximum-likelihood method (Figure 2).

### Pathogenicity test

Twelve days after inoculation, typical FHB symptoms were visible on the inoculated plants, whereas the control plants remained asymptomatic. The pathogenicity test was repeated three times with similar results. Pure cultures were re-isolated from diseased spikes and confirmed to be *F. avenaceum* based on the morphological and molecular methods mentioned above (ITS region, EF-1α and RPB-2 sequences).

### Utilization of different carbon and nitrogen sources

**FIGURE 3:**
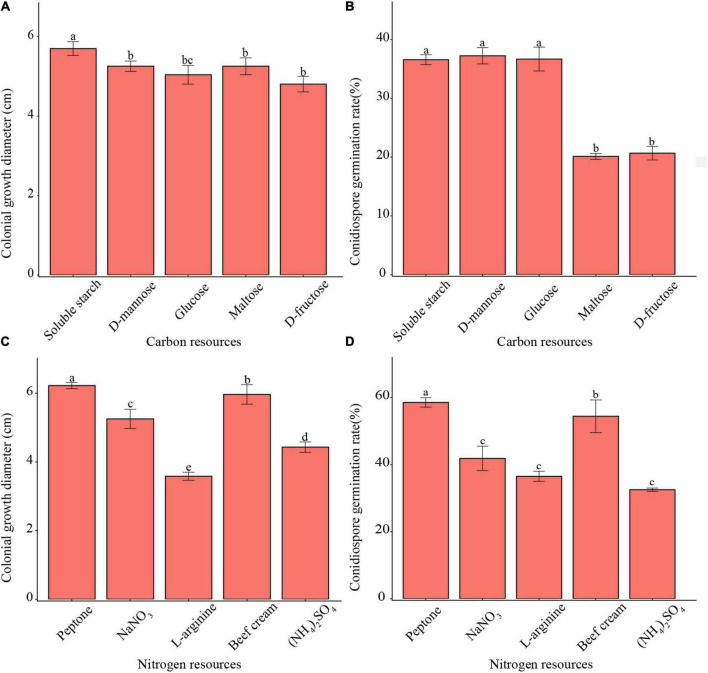
Effects of carbon and nitrogen sources on mycelium growth and conidia germination of *F. avenaceum* Charlie 779. Colonial growth diameter on different carbon sources **(A)**, conidiospore germination rate on different carbon sources **(B)**, colonial growth diameter on different nitrogen sources **(C)**, conidiospore germination rate on different nitrogen sources **(D)**. Different lowercase letters indicate significant differences (*P* < 0.05). Data are mean ± SD (*n* = 3).

All tested carbon sources enabled mycelium growth and conidia germination (Figure 3). The pathogen showed faster mycelium growth on soluble starch than on other carbon resources. Meanwhile, the pathogen demonstrated higher conidia germination rates in soluble starch, D-mannose, and glucose than in other carbon resources. Moreover, the pathogen showed both, faster mycelium growth as well as a higher conidia germination rate in peptone than with other nitrogen resources.

### Effect of temperature and photoperiod

**FIGURE 4:**
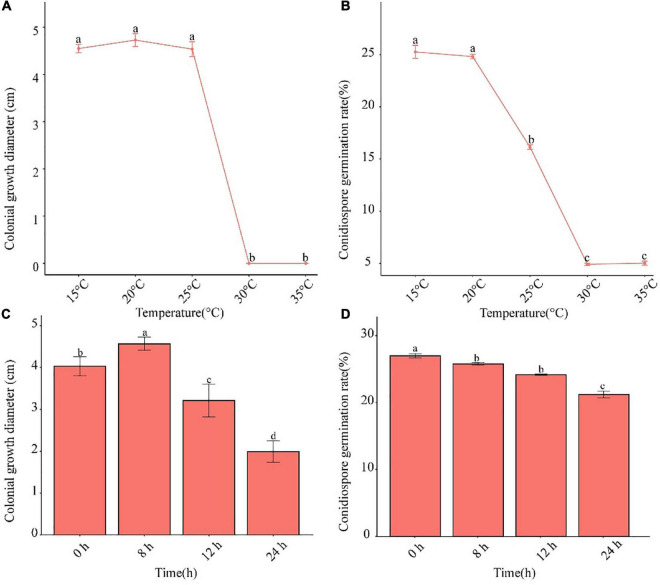
Effects of temperature and photoperiod on mycelium growth and conidia germination of *F. avenaceum* Charlie 779. Colonial growth diameter under different temperatures **(A)**, conidiospore germination rate under different temperatures **(B)**, colonial growth diameter under different photoperiods **(C)**, conidiospore germination rate under different photoperiods **(D)**. Different lowercase letters indicate significant differences (*P* < 0.05). Data are mean ± SD (*n* = 3).

The pathogen was able to grow in the temperature range of 15–25°C (Figures 4A,B). The conidia germination rate decreased after 25°C. When the temperature was higher than 30°C, neither mycelium nor conidia grew. Alternating 8-h light and 16-h dark was shown to be conducive to the growth of mycelia, and complete darkness facilitated conidia germination (Figures 4C,D).

### Effect of irradiation with ultraviolet light

**FIGURE 5:**
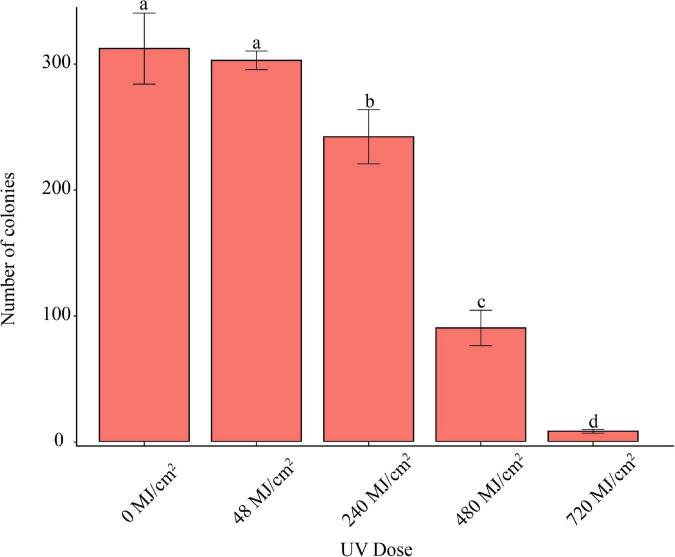
Effect of UVC irradiation on *F. avenaceum* Charlie 779. Different lowercase letters indicate significant differences (*P* < 0.05). Data are mean ± SD (*n* = 3).

UVC Irradiation for 1 min at the wave length of 254 nm, 48 MJ/cm^2^, did not inhibit the pathogen’s conidia germination (Figure 5). However, UVC Irradiation longer than 5 min, 240 MJ/cm^2^, significantly inhibited conidia germination.

### Fungicide assays

The representative isolate *F. avenaceum* Charlie 779 showed different sensitivity to the seven selected fungicides (Table 2). Among the seven tested fungicides, carbendazim showed the highest inhibition rate, with an EC_50_ (concentration for 50% of maximal effect) value of 2.1 mg/L. This was followed by propiconazole, which also showed an inhibitory effect against *F. avenac*eum, with EC_50_ values of 2.6 mg/L. Moderate effects were observed with myclobutanil and pyrimethanil, which showed EC_50_ values of 24.6 and 79.2 mg/L, respectively. In contrast, thiram, mancozeb and hymexazol showed low inhibition rates against *F. avenaceum* Charlie 779 with EC_50_ values of 136, 21, and 251 mg/L, respectively. Moreover, according to the regression equation, the slope of propiconazole was the largest at 4.8, and that of mancozeb was the smallest at 2.1. This indicated that *F. avenaceum* was most sensitive to propiconazole and the least sensitive to mancozeb.

**TABLE 2:** Inhibitory effect of seven fungicides on *F. avenaceum* Charlie 779.

Fungicides	Toxic regression equation	EC_50_(mg⋅L^–1^)	*r*	95% Confidence intervals
40% propiconazole EC	*y* = 4.7759 + 0.5465x	2.5704 ± 0.15	0.9468	1.0801–6.1168
12.5% myclobutanil EC	*y* = 4.1182 + 0.6339x	24.6029 ± 0.16	0.9768	15.7675–38.3892
40% pyrimethanil SC	*y* = 2.5944 + 1.2668x	79.2346 ± 0.21	0.9679	54.3629–115.4853
50% thiram WP	*y* = 2.7312 + 1.0622x	136.4882 ± 0.31	0.9579	85.5265–217.8158
80% mancozeb WP	*y* = 1.2486 + 1.5519x	261.3862 ± 0.28	0.9292	110.1362–620.3476
50% carbendazim WP	*y* = 4.3674 + 1.9163x	2.1385 ± 0.12	0.9933	1.8629–2.4522
70% hymexazol WP	*y* = 1.2732 + 1.5535x	250.6388 ± 0.33	0.9825	167.2725–375.5539

## Discussion

Wheat is an important nutritional resource in Tibet, which is located in high-altitude areas of Southwestern China. In this study, we identified *F. avenaceum* as the causal agent of FHB in Tibet based on morphological and molecular characterization, as well as pathogenicity tests. To our knowledge, this is the first report of *F. avenaceum* causing wheat FHB in Tibet.

In Northern and Central Europe, *F. avenaceum* was already reported as the causative pathogen of wheat FHB (Uhlig et al., 2007; Giraud et al., 2010; Anas et al., 2020). The pathogen may thrive on host crop residues during the inter-cropping season in the form of mycelia, perithecia, or chlamydospores, and then spread to wheat *via* air (Wegulo et al., 2015). Moreover, environmental and nutritional factors like temperature, light, and carbon resource, nitrogen fertilization are presumed to greatly influence FHB infections in wheat (Hofer et al., 2016; Moreno-Amores et al., 2020; Costa et al., 2021). Low air temperature was previously shown to increase disease severity of FHB in Poland (Okorski et al., 2022). In some areas, the predominant causal agent was *F. avenaceum*. This is consistent with our study in which we isolated the pathogen, *F. avenaceum*, from Linzhi, Tibet, where the average temperature is 15.0°C in June, and 15.2°C in July [data from China Meteorological Data Service Center (CMDSC)^1^ ]. Furthermore, we characterized the influence of temperature on the pathogen, *F. avenaceum* Charlie 779, mycelium growth and conidia germination, and found 15–20°C was its optimal temperature range. Nitrogen fertilization was previously shown to influence the occurrence of FHB in wheat. Reasonable application reduces occurrence of FHB in wheat by influencing canopy characteristics and plant physiology (Hofer et al., 2016). In contrast, high nitrogen availability decreased the activity of antioxidative enzymes, and may thus increase the incidence and intensity of FHB (Matić et al., 2021). In our study, (NH_4_)SO_2_, as the sole nitrogen resource, enhanced mycelium growth and conidia germination of *F. avenaceum*. Therefore, we suggest that the applied amount of fertilizers that contain (NH_4_)SO_2_ as the major nitrogen source should be carefully considered in wheat fields previously affected by *F. avenaceum* in Tibet, China. Carbon dioxide (CO_2_) may also influence wheat quality and FHB prevalence. Hay et al. (2022) found at elevated CO_2_, more FHB resistant cultivars having greater changes in nutrient content, including protein, starch, phosphorus, and magnesium. Hence, for future wheat breeding, cultivars that can maintain both nutritional integrity and FHB resistance are desired. In addition, external carbon sources could alter the biosynthesis of fungal mycotoxins. Particular in *F. avenaceum* isolates, sucrose was reported as one of the optimal sources of carbon for enniatins production (Gautier et al., 2020). In our study, we found that all tested carbon sources supported the mycelium growth and conidia germination. However, the pathogen showed both higher mycelium growth and conidia germination on soluble starch than on other carbon resources. For future studies, it will be important to investigate how other carbon resources, except for CO_2_, may influence the disease prevalence and to explore how they could be considered in wheat breading. Meanwhile, reducing the input of carbon resources that support FHB development and mycotoxin production could provide new solutions for sustainable management of the disease.

Photoperiod was reported as an important environmental factor that may impact the biological characteristics of phytopathogens, as well as their pathogenesis (Costa et al., 2021; Macioszek et al., 2021). In the present study, we found that alternating 8-h light and 16-h cycles dark was conducive to the growth of *F. avenaceum* Charlie 779 mycelia, and full darkness facilitated its conidia germination. This was different with *F. fujikuroi* FKMC 1995, where light did not significantly affect its mycelial growth and conidia germination (Costa et al., 2021). Similarly, Zhu et al. (2013) demonstrated that mycelial growth and conidia germination of *F. oxysporum* F0112 was not affected by the photoperiod. However, for some *F. oxysporum* isolates, complete darkness significantly elicited mycelium growth and/or conidia germination (Pan et al., 2011; Jin et al., 2019). Therefore, within *Fusarium* spp., strain-specific responses of mycelial growth and conidia germination to photoperiod may widely exist.

For some pathogens like the powdery mildew causative agent, *Blumeria graminis*, UV irradiation was shown to reduce infection and proliferation by restricting prepenetration processes (Zhu et al., 2019a). However, for pathogens like *F. oxysporum*, which are located inside the host tissue, when their filament is established, the pathogen is protected from natural irradiation (Milo-Cochavi et al., 2019). In our study, we found that nearly 100 times stronger UVC irradiation than the local natural condition in Linzhi (48 MJ/cm^2^) could not significantly inhibit the mycelium growth and conidia germination of *F. avenaceum*. The results indicated that high UVC irradiation is not affecting FHB caused by *F*. *avenaceum*.

**FIGURE 6:**
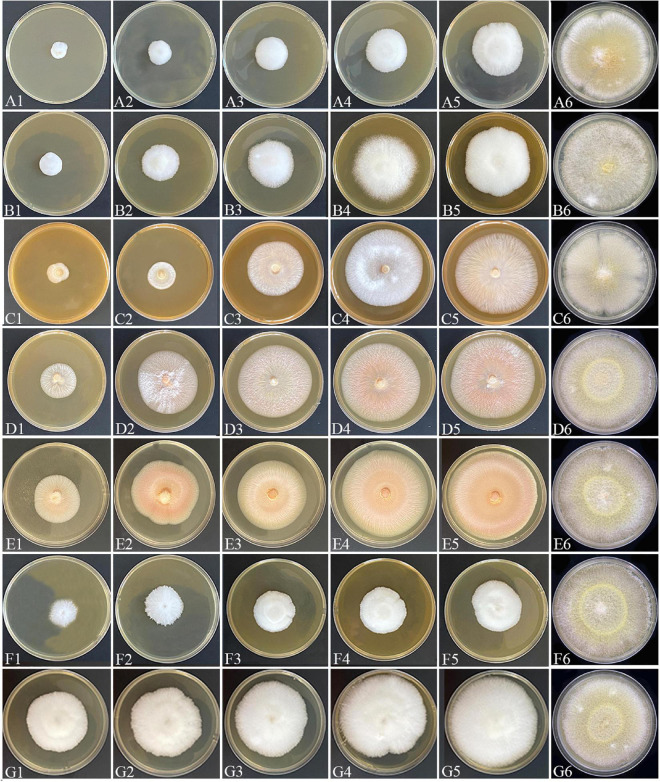
Mycelial growth of *F. avenaceum* Charlie 779 on PDA plates incubated for 7 days in the absence (CK) or presence of different concentrations of propiconazole (A1: 100 μg/mL, A2: 50 μg/mL, A3: 10 μg/mL, A4: 5 μg/mL, A5: 1 μg/mL, A6: CK), myclobutanil (B1: 100 μg/mL, B2: 50 μg/mL, B3: 10 μg/mL, B4: 5 μg/mL, B5: 1 μg/mL, B6: CK), pyrimethanil (C1: 1000 μg/mL, C2: 500 μg/mL, C3: 250 μg/mL, C4: 125 μg/mL, C5: 62.5 μg/mL, C6: CK), thiram (D1: 500 μg/mL, D2: 300 μg/mL, D3: 150 μg/mL, D4: 30 μg/mL, D5: 15 μg/mL, D6: CK), mancozeb (E1: 300 μg/mL, E2: 100 μg/mL, E3: 50 μg/mL, E4: 25 μg/mL, E5: 12.5 μg/mL, E6: CK), carbendazim (F1: 6 μg/mL, F2: 3 μg/mL, F3: 1.5 μg/mL F4: 0.75 μg/mL, F5: 0.38 μg/mL, F6: CK), and hymexazol (G1: 200 μg/mL, G2: 100 μg/mL, G3: 50 μg/mL, G4: 25 μg/mL, G5: 12.5 μg/mL, G6: CK).

Chemical control represents one of the main measures for the prevention and control of wheat FHB (Dweba et al., 2017). Therefore, we conducted an *in vitro* screening of potentially effective fungicides against *F. avenaceum* Charlie 779 (Figure 6). Among the seven tested pesticides, carbendazim and propiconazole showed the best inhibition rates, with an EC_50_ value of 2.1 and 2.6 mg/L, respectively. The results were in accordance with previous studies which showed carbendazim and propiconazole are effective against the FHB causal agent *F. graminearum* (Klix et al., 2007; Liu et al., 2014). Doohan et al. (1999) reported that pyrimethanil showed no reduction of FHB symptoms. In our study, pyrimethanil in our study showed moderate inhibitory effects against *F. avenaceum*. Generally, thiram and mancozeb were shown to inhibit mycelial growth of FHB isolates when combined with other fungicides (Zhang et al., 2009; Park et al., 2012; Mengesha et al., 2022). However, these compounds showed poor inhibition rates against *F. avenaceum* in the present study when they were separately applied *in vitro.* For myclobutanil and hymexazol, no reports are available yet regarding their effect on FHB isolates. Interestingly, we found that myclobutanil showed a relatively high inhibitory effect against *F. avenaceum*. In contrast, hymexazol showed poor effects. Our study demonstrated the potential of carbendazim, propiconazole, and myclobutanil for chemical control of wheat FHB caused by *F. avenaceum* in Tibet.

General adaptability and environmental factors may contribute to the development of fungicide resistance in *F. avenaceum* as commonly observed with other fungal pathogens (Sun et al., 2020; Chen et al., 2021). Considering that the isolates described in the present study were obtained from a unique environment, their biological characteristic may differ from other isolates from common cultivation areas and they might be either more or less prone to resistance development. Therefore, the relationships and/or correlations between FHB incidence, control effect, the pathogen’s biological characteristics, and the development of fungicide resistance should be further examined.

Overall, our study provides new insights into the occurrence and causal agent of wheat FHB in Tibet, China as well as the pathogen’s biological characteristics and promising candidate fungicides for its management. As discussed above, further studies will be needed to develop effective and sustainable control approaches against FHB in Tibet.

## Data availability statement

The datasets presented in this study can be found in online repositories. The names of the repository/repositories and accession number(s) can be found in the article/Supplementary material.

## Author contributions

XC and TC designed the experiments. XT, GY, GZ, XG, PC, BY, WW, and DJ performed the experiments and drafted the manuscript. XT and XC analyzed data. TC, MP, IB, and QM conducted visualization and proofreading of the manuscript. All authors approved the manuscript for submission.

## Footnotes

1
http://101.201.172.75:8888/dataGis/static/visual/#/visual
