# Supplementary material for: Biological characterization and in vitro fungicide screenings of a new causal agent of wheat Fusarium head blight in Tibet, China
Source: Front Microbiol. 2022 Aug 5;13:941734. doi: 10.3389/fmicb.2022.941734 (PMC9389214; doi:10.3389/fmicb.2022.941734)
Supplement: Supplementary file 1 [file Data_Sheet_1.DOCX]

**Supplementary File 1**

1. *Fusarium avenaceum* Charlie 779 ITS region (accession number: MZ049674)

TCTAGCATCTACTGATCCGAGGTCACATTCAGAAGTTGGGGTTTTACGGCATGGCCGCGCCGCGTTCCAGTTGCGAGGTGTTAGCTACTACGCAATGGAGGCTGCAGCGAGACCGCCAATGTATTTCGGGGGCGGCACCGCCAGAAGGCAGAGCCGATCCCCAACACCAAACCCGGGGGCTTGAGGGTTGAAATGACGCTCGAACAGGCATGCCCGCCGGAATACCAGCGGGCGCAATGTGCGTTCAAAGATTCGATGATTCACTGAATTCTGCAATTCACATTACTTATCGCATTTTGCTGCGTTCTTCATCGATGCCAGAACCAAGAGATCCGTTGTTGAAAGTTTTGATTTATTTGTTTGTTTTACTCAGAAGTTACAATAAGAAACATTAGAGTTTGGGTCCTCTGGCGGGCCGTCCCGTTTTACGGGGCGCGGGCTGATCCGCCGAGGCAACATTAAGGTATGATCAGTAGATGCTAGA

1. *Fusarium avenaceum* Charlie 779[elongation factor 1a (tef1) gene, partial cds](https://www.ncbi.nlm.nih.gov/nuccore/JQ429374.1). (accession number: MZ053464 )

AATGCGTTCGATACGCGCGCTCCATCGATTCCCACGACTCGCTCCCTCATTCGAAACGCATTCATTACCCCGCTCAAGTCCGAAAATTTTGCGGTGCGACCGTGATTTTTTTTGGTGGGGTATCTTACCCCGCCACTCGAGTGACGGATGCGCTTGCCCTGTTCCCACAAAACCTTACCACACTGTCGCGCACTATGTCTTGCAGTCACTAACCACTGGACAATAGGAAGCCGCCGAGCTCGGAAAGGGTTCCTTCAAGTACGCCTGGGTTCTTGACAAGCTCAAAGCCGAGCGTGAGCGTGGTATCACCATTGATATCGCTCTCTGGAAGTTCGAGACTCCTCGCTACTATGTCACCGTCATTGGTATGTTGTCACTGTCTCACACCACCATGCCTTCATCATGCTAACATTCCTCTCAGATGCCCCCGGTCATCGTGATTTCATCAAGAACATGATTACTGGTACTTCCCAGGCTGATTGTGCCATTCTCATCATTGCCGCCGGTACGCATT

3）*Fusarium avenaceum* Charlie 779 DNA-directed RNA polymerase II core subunit (RPB2) gene partial cds. (accession number: MZ053465)

ATGGGATTTACCCTAGTAACTCGCTCTGTTGGCGAGCTCTTCCGTGGTATTATCCGCAGGATGAACACTGAGCTGTCCAACTACCTAAAGCGCTGTGTTGAAAGCAACAGACATTTCAACTTGGCTGTCGCTATCAAGCCAGGAACATTGTCCAATGGTCTGAAGTATTCGCTGGCCACTGGTAACTGGGGAGATCAAAAGAAGGCAGCAAGCTCCACAGCTGGTGTCTCTCAGGTGTTGAACAGATATACCTTCGCTTCTACTCTGTCTCATTTGCGACGTACCAACACCCCCATCGGACGAGATGGTAAGCTGGCCAAACCCCGTCAGCTACACAACACCCATTGGGGTTTGGTGTGTCCTGCAGAAACGCCTGAGGGACAGGCTTGTGGTCTGGTCAAGAACTTGTCTCTGATGTGTTACGTGAGTGTGGGCTCTCCTGCTGATCCTCTGATTGACTTCATGATCCACAGAGGTATGGAAGTGGTTGAGGAGTATGAGCCAACAAGATACCCACACGCTACCAAGATTTTCGTCAACGGTAGCTGGGTTGGTGTTCACTCTGACCCCAAGCATCTTGTTCACCAAGTTTTGTCCACCCGACGAAAGAATGTCGTCCAATTCGAAGTGTCACTTGTTCGTGATATTCGAGACCGAGAATTCAAGATCTTCTCTGATGCAGGCAGAGTCATGAGACCGGTCTTTACAGTACAGCAGGAGGATGACGACGAGACTGGTATTCAGAAGGGACAGCTTATACTGACCAAGGAGCTGGTTACCAAGCTCGCCCAAGAGCAGGCGGAGCCATCGGATGACCCATCAGAGAAGCTTGGCTGGGAGGGTCTTGTTCGCGCTGGAGTTATCGAGTATCTCGATGCCGAGGAAGAAGAAACGGCCATGATCTGCATGACGCCCGAGGATCTCGAACTTTACCGCGAGCAAAAGATCGACGAGGCGACCCTCACAGTGGAAGAGAAGTGGAAGAGAGGCAGGCTAAGCAGGAGGCTGAGAAGAGAGAACAAGAGGAAGAACGCAACAAGAGATTGAAGACAAAGGTCAATCCTACGACTCACGTGTACACACATTGTGAGATTCATCCCAGTATGATTCTTGGTATTTGTGCCAGTATCATTCCCTTCCCTGATCACAACCAGGTATGTCAGGACGCTTAATTTTGGTTGATTACGATCGAACGACGCACATATGATCGACCCGTGTGCAC

4）*Fusarium avenaceum* Charlie 782 ITS region (accession number: [ON805846](https://www.ncbi.nlm.nih.gov/nuccore/ON805846))

GTGAACCTGCGGAGGGATCATTACCGAGTTTACAACTCCCAAACCCCTGTGAACATACCTTAATGTTGCCTCGGCGGATCAGCCCGCGCCCCGTAAAACGGGACGGCCCGCCAGAGGACCCAAACTCTAATGTTTCTTATTGTAACTTCTGAGTAAAACAAACAAATAAATCAAAACTTTCAACAACGGATCTCTTGGTTCTGGCATCGATGAAGAACGCAGCAAAATGCGATAAGTAATGTGAATTGCAGAATTCAGTGAATCATCGAATCTTTGAACGCACATTGCGCCCGCTGGTATTCCGGCGGGCATGCCTGTTCGAGCGTCATTTCAACCCTCAAGCCCCCGGGTTTGGTGTTGGGGATCGGCTCTGCCTTCTGGCGGTGCCGCCCCCGAAATACATTGGCGGTCTCGCTGCAGCCTCCATTGCGTAGTAGCTAACACCTCGCAACTGGAACGCGGCGCGGCCATGCCGTAAAACCCCAACTTCTGAATGTTGACCTCGGATCAGGTAGGAATACCCGCTGAACTTAAGCATATCAAAAAGGCGGAGGA

5）*Fusarium avenaceum* Charlie 782[elongation factor 1a (tef1) gene, partial cds](https://www.ncbi.nlm.nih.gov/nuccore/JQ429374.1). (accession number: ON833468 )

GGTTAGTCAATATCCCTTCGATTACGCGCGCTCCCATCGATTCCCACGACTCGCTCCCTCATTCGAAACGCATTCATTACCCCGCTCAAGTCCGAAAATTTTGCGGTGCGACCGTGATTTTTTTTGGTGGGGTATCTTACCCCGCCACTCGAGTGACGGATGCGCTTGCCCTGTTCCCACAAAACCTTACCACACTGTCGCGCACTATGTCTTGCAGTCACTAACCACTGGACAATAGGAAGCCGCCGAGCTCGGAAAGGGTTCCTTCAAGTACGCCTGGGTTCTTGACAAGCTCAAAGCCGAGCGTGAGCGTGGTATCACCATTGATATCGCTCTCTGGAAGTTCGAGACTCCTCGCTACTATGTCACCGTCATTGGTATGTTGTCACTGTCTCACACCACCATGCCTTCATCATGCTAACATTCCTCTCAGATGCCCCCGGTCATCGTGATTTCATCAAGAACATGATTACTGGTACTTCCCAGGCTGATTGTGCCATTCTCATCATTGCCGCCGGTACTGGTGAGTTCGAGGCT

6）*Fusarium avenaceum* Charlie 782 DNA-directed RNA polymerase II core subunit (RPB2) gene partial cds. (accession number: ON833471)

GACAGATATACCTTCGCTTCTACTCTGTCTCATTTGCGACGTACCAACACCCCCATCGGACGAGATGGTAAGCTGGCCAAACCCCGTCAGCTACACAACACCCATTGGGGTTTGGTGTGTCCTGCAGAAACGCCTGAGGGACAGGCTTGTGGTCTGGTCAAGAACTTGTCTCTGATGTGTTACGTGAGTGTGGGCTCTCCTGCTGATCCTCTGATTGACTTCATGATCCACAGAGGTATGGAAGTGGTTGAGGAGTATGAGCCAACAAGATACCCACACGCTACCAAGATTTTCGTCAACGGTAGCTGGGTTGGTGTTCACTCTGACCCCAAGCATCTTGTTCACCAAGTTTTGTCCACCCGACGAAAGAATGTCGTCCAATTCGAAGTGTCACTTGTTCGTGATATTCGAGACCGAGAATTCAAGATCTTCTCTGATGCAGGCAGAGTCATGAGACCGGTCTTTACAGTACAGCAGGAGGATGACGACGAGACTGGTATTCAGAAGGGACAGCTTATACTGACCAAGGAGCTGGTTACCAAGCTCGCCCAAGAGCAGGCGGAGCCATCGGATGACCCATCAGAGAAGCTTGGCTGGGAGGGTCTTGTTCGCGCTGGAGTTATCGAGTATCTCGATGCCGAGGAAGAAGAAACGGCCATGATCTGCATGACGCCCGAGGATCTCGAACTTTACCGCGAGCAAAAGATCGACGAGGCGACCCTCACAGTGGAAGAGAGGCAGGCTAAGCAGGAGGCTGAGAAGAGAGAACAAGAGGAAGAACGCAACAAGAGATTGAAGACAAAGGTCAATCCTACGACTCACGTGTACACACATTGTGAGATTCATCCCAGTATGATTCTTGGTATTTGTGCCAGTATCATTCCCTTCCCTGATCACAACCAGGTATGTCAGGACGCTTAATTTTGGTTGATCCTTACTAACAACACACA

7）*Fusarium avenaceum* Charlie 788 ITS region (accession number: [ON805862](https://www.ncbi.nlm.nih.gov/nuccore/ON805862))

CGTAGGGTGAACCTGCGGAGGGATCATTACCGAGTTTACAACTCCCAAACCCCTGTGAACATACCTTAATGTTGCCTCGGCGGATCAGCCCGCGCCCCGTAAAACGGGACGGCCCGCCAGAGGACCCAAACTCTAATGTTTCTTATTGTAACTTCTGAGTAAAACAAACAAATAAATCAAAACTTTCAACAACGGATCTCTTGGTTCTGGCATCGATGAAGAACGCAGCAAAATGCGATAAGTAATGTGAATTGCAGAATTCAGTGAATCATCGAATCTTTGAACGCACATTGCGCCCGCTGGTATTCCGGCGGGCATGCCTGTTCGAGCGTCATTTCAACCCTCAAGCCCCCGGGTTTGGTGTTGGGGATCGGCTCTGCCTTCTGGCGGTGCCGCCCCCGAAATACATTGGCGGTCTCGCTGCAGCCTCCATTGCGTAGTAGCTAACACCTCGCAACTGGAACGCGGCGCGGCCATGCCGTAAAACCCCAACTTCTGAATGTTGACCTCGGATCAGGTAGGAATACCCGCTGAACTTAAGCATATCATAAGGCGGGAGGA

8）*Fusarium avenaceum* Charlie 788 [elongation factor 1a (tef1) gene, partial cds](https://www.ncbi.nlm.nih.gov/nuccore/JQ429374.1). (accession number: ON833469)

AGAAGGTTAGTCAATATCCCTTCGATTACGCGCGCTCCCATCGATTCCCACGACTCGCTCCCTCATTCGAAACGCATTCATTACCCCGCTCAAGTCCGAAAATTTTGCGGTGCGACCGTGATTTTTTTTGGTGGGGTATCTTACCCCGCCACTCGAGTGACGGATGCGCTTGCCCTGTTCCCACAAAACCTTACCACACTGTCGCGCACTATGTCTTGCAGTCACTAACCACTGGACAATAGGAAGCCGCCGAGCTCGGAAAGGGTTCCTTCAAGTACGCCTGGGTTCTTGACAAGCTCAAAGCCGAGCGTGAGCGTGGTATCACCATTGATATCGCTCTCTGGAAGTTCGAGACTCCTCGCTACTATGTCACCGTCATTGGTATGTTGTCACTGTCTCACACCACCATGCCTTCATCATGCTAACATTCCTCTCAGATGCCCCCGGTCATCGTGATTTCATCAAGAACATGATTACTGGTACTTCCCAGGCTGATTGTGCCATTCTCATCATTGCCGCCGGTACTGGTGAGTTCGAGGCTGGTA

9）*Fusarium avenaceum* Charlie 788 DNA-directed RNA polymerase II core subunit (RPB2) gene partial cds. (accession number: ON833472)

TACCTTCGCTTCTACTCTGTCTCATTTGCGACGTACCAACACCCCCATCGGACGAGATGGTAAGCTGGCCAAACCCCGTCAGCTACACAACACCCATTGGGGGTTTGGTGTGTCCTGCAGAAACGCCTGAGGGACAGGCTTGTGGTCTGGTCAAGAACTTGTCTCTGATGTGTTACGTGAGTGTGGGCTCTCCTGCTGATCCTCTGATTGACTTCATGATCCACAGAGGTATGGAAGTGGTTGAGGAGTATGAGCCAACAAGATACCCACACGCTACCAAGATTTTCGTCAACGGTAGCTGGGTTGGTGTTCACTCTGACCCCAAGCATCTTGTTCACCAAGTTTTGTCCACCCGACGAAAGAATGTCGTCCAATTCGAAGTGTCACTTGTTCGTGATATTCGAGACCGAGAATTCAAGATCTTCTCTGATGCAGGCAGAGTCATGAGACCGGTCTTTACAGTACAGCAGGAGGATGACGACGAGACTGGTATTCAGAAGGGACAGCTTATACTGACCAAGGAGCTGGTTACCAAGCTCGCCCAAGAGCAGGCGGAGCCATCGGATGACCCATCAGAGAAGCTTGGCTGGGAGGGTCTTGTTCGCGCTGGAGTTATCGAGTATCTCGATGCCGAGGAAGAAGAAACGGCCATGATCTGCATGACGCCCGAGGATCTCGAACTTTACCGCGAGCAAAAGATCGACGAGGCGACCCTCACAGTGGAAGAGAGGCAGGCTAAGCAGGAGGCTGAGAAGAGAGAACAAGAGGAAGAACGCAACAAGAGATTGAAGACAAAGGTCAATCCTACGACTCACGTGTACACACATTGTGAGATTCATCCCAGTATGATTCTTGGKATTTGTGCCAGTATCATTCCCTTCCCTGATCACAACCAGGTATGTCAGGACGCTTAATTTTGGTTGATCCTTACTAACAACACAC

10）*Fusarium avenaceum* Charlie 789 ITS region (accession number: [ON819345](https://www.ncbi.nlm.nih.gov/nuccore/ON819345))

TATGATATGCTTAAGTTCAGCGGGTATTCCTACCTGATCCGAGGTCAACATTCAGAAGTTGGGGTTTTACGGCATGGCCGCGCCGCGTTCCAGTTGCGAGGTGTTAGCTACTACGCAATGGAGGCTGCAGCGAGACCGCCAATGTATTTCGGGGGCGGCACCGCCAGAAGGCAGAGCCGATCCCCAACACCAAACCCGGGGGCTTGAGGGTTGAAATGACGCTCGAACAGGCATGCCCGCCGGAATACCAGCGGGCGCAATGTGCGTTCAAAGATTCGATGATTCACTGAATTCTGCAATTCACATTACTTATCGCATTTTGCTGCGTTCTTCATCGATGCCAGAACCAAGAGATCCGTTGTTGAAAGTTTTGATTTATTTGTTTGTTTTACTCAGAAGTTACAATAAGAAACATTAGAGTTTGGGTCCTCTGGCGGGCCGTCCCGTTTTACGGGGCGCGGGCTGATCCGCCGAGGCAACATTAAGGTATGTTCACAGGGGTTTGGGAGTTGTAAACTCGGTAATGATCCCTCCGCAGGTTCCCCTTAACGGAGGGATCATTTACCGAGTTTACAACTCCCAAACCCCTGTGAACATACCTTAATGTTGCCTCGGCGGATCAGCCCGCGCCC

1. *Fusarium avenaceum* Charlie 789 [elongation factor 1a (tef1) gene, partial cds](https://www.ncbi.nlm.nih.gov/nuccore/JQ429374.1). (accession number: ON833470)

GAGAAGGTTAGTCAATATCCCTTCGATTACGCGCGCTCCCATCGATTCCCACGACTCGCTCCCTCATTCGAAACGCATTCATTACCCCGCTCAAGTCCGAAAATTTTGCGGTGCGACCGTGATTTTTTTTGGTGGGGTATCTTACCCCGCCACTCGAGTGACGGATGCGCTTGCCCTGTTCCCACAAAACCTTACCACACTGTCGCGCACTATGTCTTGCAGTCACTAACCACTGGACAATAGGAAGCCGCCGAGCTCGGAAAGGGTTCCTTCAAGTACGCCTGGGTTCTTGACAAGCTCAAAGCCGAGCGTGAGCGTGGTATCACCATTGATATCGCTCTCTGGAAGTTCGAGACTCCTCGCTACTATGTCACCGTCATTGGTATGTTGTCACTGTCTCACACCACCATGCCTTCATCATGCTAACATTCCTCTCAGATGCCCCCGGTCATCGTGATTTCATCAAGAACATGATTACTGGTACTTCCCAGGCTGATTGTGCCATTCTCATCATTGCCGCCGGTACTGGTGGAGTTCGAGGCTGGC

1. *Fusarium avenaceum* Charlie 789 DNA-directed RNA polymerase II core subunit (RPB2) gene partial cds. (accession number: ON833473)

ATACCTTCGCTTCTACTCTGTCTCATTTGCGACGTACCAACACCCCCATCGGACGAGATGGTAAGCTGGCCAAACCCCGTCAGCTATTCACAACACCCATTGGGGKTTGGTGTGTCCTGCAGAAACGCCTGAGGGACAGGCTTGTGGTCTGGTCAAGAACTTGTCTCTGATGTGTTACGTGAGTGTGGGCTCTCCTGCTGATCCTCTGATTGACTTCATGATCCACAGAGGTATGGAAGTGGTTGAGGAGTATGAGCCAACAAGATACCCACACGCTACCAAGATTTTCGTCAACGGTAGCTGGGTTGGTGTTCACTCTGACCCCAAGCATCTTGTTCACCAAGTTTTGTCCACCCGACGAAAGAATGTCGTCCAATTCGAAGTGTCACTTGTTCGTGATATTCGAGACCGAGAATTCAAGATCTTCTCTGATGCAGGCAGAGTCATGAGACCGGTCTTTACAGTACAGCAGGAGGATGACGACGAGACTGGTATTCAGAAGGGACAGCTTATACTGACCAAGGAGCTGGTTACCAAGCTCGCCCAAGAGCAGGCGGAGCCATCGGATGACCCATCAGAGAAGCTTGGCTGGGAGGGTCTTGTTCGCGCTGGAGTTATCGAGTATCTCGATGCCGAGGAAGAAGAAACGGCCATGATCTGCATGACGCCCGAGGATCTCGAACTTTACCGCGAGCAAAAGATCGACGAGGCGACCCTCACAGTGGAAGAGAGGCAGGCTAAGCAGGAGGCTGAGAAGAGAGAACAAGAGGAAGAACGCAACAAGAGATTGAAGACAAAGGTCAATCCTACGACTCACGTGTACACACATTGTGAGATTCATCCCAGTATGATTCCTTGGTATTTGTGCCAGTATCATTCCCTTCCCTGATCACAACCAGGTATGTCAGGACGCTTAATTTTGGTTGATCCTTACTAACAAC

13 >*Fusarium avenaceum* isolate Charlie 790 ITS region (accession number: ON847358)

TATGCTTAAGTTCAGCGGGTATTCCTACCTGATCCGAGGTCAACATTCAGAAGTTGGGGTTTTACGGCATGGCCGCGCCGCGTTCCAGTTGCGAGGTGTTAGCTACTACGCAATGGAGGCTGCAGCGAGACCGCCAATGTATTTCGGGGGCGGCACCGCCAGAAGGCAGAGCCGATCCCCAACACCAAACCCGGGGGCTTGAGGGTTGAAATGACGCTCGAACAGGCATGCCCGCCGGAATACCAGCGGGCGCAATGTGCGTTCAAAGATTCGATGATTCACTGAATTCTGCAATTCACATTACTTATCGCATTTTGCTGCGTTCTTCATCGATGCCAGAACCAAGAGATCCGTTGTTGAAAGTTTTGATTTATTTGTTTGTTTTACTCAGAAGTTACAATAAGAAACATTAGAGTTTGGGTCCTCTGGCGGGCCGTCCCGTTTTACGGGGCGCGGGCTGATCCGCCGAGGCAACATTAAGGTATGTTCACAGGGGTTTGGGAGTTGTAAACTCGGTAATGATCCCTCCGCA

14 >*Fusarium avenaceum* isolate Charlie 790 [elongation factor 1a (tef1) gene, partial cds](https://www.ncbi.nlm.nih.gov/nuccore/JQ429374.1). (accession number:ON868916)

AAGGTTAGTCAATATCCCTTCGATTACGCGCGCTCCCATCGATTCCCACGACTCGCTCCCTCATTCGAAACGCATTCATTACCCCGCTCAAGTCCGAAAATTTTGCGGTGCGACCGTGATTTTTTTTGGTGGGGTATCTTACCCCGCCACTCGAGTGACGGATGCGCTTGCCCTGTTCCCACAAAACCTTACCACACTGTCGCGCACTATGTCTTGCAGTCACTAACCACTGGACAATAGGAAGCCGCCGAGCTCGGAAAGGGTTCCTTCAAGTACGCCTGGGTTCTTGACAAGCTCAAAGCCGAGCGTGAGCGTGGTATCACCATTGATATCGCTCTCTGGAAGTTCGAGACTCCTCGCTACTATGTCACCGTCATTGGTATGTTGTCACTGTCTCACACCACCATGCCTTCATCATGCTAACATTCCTCTCAGATGCCCCCGGTCATCGTGATTTCATCAAGAACATGATTACTGGTACTTCCCAGGCTGATTGTGCCATTCTCATCATTGCCGCCGGTACTGGTGGAGTTCG

15 >*Fusarium avenaceum* isolate Charlie 790 DNA-directed RNA polymerase II core subunit (RPB2) gene partial cds. (accession number: ON868917)

CCTTCGCTTCTACTCTGTCTCATTTGCGACGTACCAACACCCCCATCGGACGAGATGGTAAGCTGGCCAAACCCCGTCAGCTATTCACAACACCCATTGGGGTTTGGTGTGTCCTGCAGAAACGCCTGAGGGACAGGCTTGTGGTCTGGTCAAGAACTTGTCTCTGATGTGTTACGTGAGTGTGGGCTCTCCTGCTGATCCTCTGATTGACTTCATGATCCACAGAGGTATGGAAGTGGTTGAGGAGTATGAGCCAACAAGATACCCACACGCTACCAAGATTTTCGTCAACGGTAGCTGGGTTGGTGTTCACTCTGACCCCAAGCATCTTGTTCACCAAGTTTTGTCCACCCGACGAAAGAATGTCGTCCAATTCGAAGTGTCACTTGTTCGTGATATTCGAGACCGAGAATTCAAGATCTTCTCTGATGCAGGCAGAGTCATGAGACCGGTCTTTACAGTACAGCAGGAGGATGACGACGAGACTGGTATTCAGAAGGGACAGCTTATACTGACCAAGGAGCTGGTTACCAAGCTCGCCCAAGAGCAGGCGGAGCCATCGGATGACCCATCAGAGAAGCTTGGCTGGGAGGGTCTTGTTCGCGCTGGAGTTATCGAGTATCTCGATGCCGAGGAAGAAGAAACGGCCATGATCTGCATGACGCCCGAGGATCTCGAACTTTACCGCGAGCAAAAGATCGACGAGGCGACCCTCACAGTGGAAGAGAGGCAGGCTAAGCAGGAGGCTGAGAAGAGAGAACAAGAGGAAGAACGCAACAAGAGATTGAAGACAAAGGTCAATCCTACGACTCACGTGTACACACATTGTGAGATTCATCCCAGTATGATTCCTTGGTATTTGTGCCAGTATCATTCCCTTCCCTGATCACAACCAGGTATGTCAGGACGCTTAATTTTGGTTGATCC
